# Cost-effectiveness of lung volume reduction coil treatment in patients with severe emphysema: results from the 2-year follow-up crossover REVOLENS study (REVOLENS-2 study)

**DOI:** 10.1186/s12931-018-0796-x

**Published:** 2018-05-09

**Authors:** Julie Bulsei, Sylvie Leroy, Jeanne-Marie Perotin, Hervé Mal, Charles-Hugo Marquette, Hervé Dutau, Arnaud Bourdin, Jean-Michel Vergnon, Christophe Pison, Romain Kessler, Vincent Jounieaux, Mathieu Salaün, Armelle Marceau, Sylvain Dukic, Coralie Barbe, Margaux Bonnaire, Gaëtan Deslee, Isabelle Durand-Zaleski, the REVOLENS study group, Sandra Dury, Hervé Vallerand, François Lebargy, Claire Launois, Johanna Pradelli, Jonathan Benzaquen, Matthieu Buscot, Celine Sanfiorenzo, Sylvia Korzeniewski, Andrea Mazzetta, Jennifer Griffonnet, Ariane Guillemart, Demosthenes Makris, Christine Dombret, Frédérique Servin, Marie Pierre Debray, Sophie Laroumagne, Fabienne Bregeon, Carine Gomez, Philippe Astoul, Nathalie Lesavre, Jean Pierre Mallet, Anne Sophie Gamez, Philippe Tarodo, Christophe Brousse, Clément Broissin, Yoann Thibout, Fabrice Di Palma, Frédéric Costes, Amandine Briault, François Arbib, Emilie Reymond, G. Ferrettti, Michele Porzio, Benjamin Renaud-Picard, Bénédicte Toublanc, Géraldine François, Luc Thiberville, Géraldine François, Luc Thiberville, Antoine Cuvelier, Samy Lachkar, Delphine Gras, Philippe Benoit, Annick Leclainche, Damien Jolly, François Fourrier, Eric Hachulla, Nicolas Roche, Daniel Dusser

**Affiliations:** 1 AP-HP URC Eco IdF, Unité de recherche clinique en économie de la santé d’Ile de France, Paris, France; 2 Service de Pneumologie, CHU de Nice, FHU OncoAge, Université Côte d’Azur, Nice, France; 3 Service de Pneumologie, INSERM U1250, Hôpital Universitaire, Reims, France; 4 Service de Pneumologie, Hôpital Universitaire Bichat, Paris, France; 5 Service d’Oncologie Thoracique, Maladies de la Plèvre, Pneumologie Interventionnelle, Hôpital Universitaire, Marseille, France; 6 Département de Pneumologie et Addictologie, PhyMedExp, INSERM U1046, CNRS UMR, Hôpital Universitaire, Montpellier, France; 7 Service de Pneumologie, Hôpital Universitaire, Saint Etienne, France; 8 Pôle Thorax et Vaisseaux, Inserm1055, Hôpital Universitaire Grenoble Alpes, Inserm1055, Université Grenoble Alpes, Grenoble, France; 9 Service de Pneumologie, Hôpital Universitaire, Strasbourg, France; 10 Service de Pneumologie, Hôpital Universitaire, Amiens, France; 11 Service de Pneumologie, Oncologie Thoracique et Soins Intensifs Respiratoires, Hôpital Universitaire, Rouen, France; 12 Département de Pharmacovigilance, Hôpital Universitaire de Reims, Reims, France; 13 Unité d’Aide Méthodologique, Pôle Recherche et Santé Publique, Hôpital Universitaire de Reims, Reims, France; 14 Centre de Recherche et d’Investigation Clinique, Pôle Recherche et Santé Publique, Hôpital Universitaire de Reims, Reims, France

**Keywords:** Coil treatment, Severe emphysema, Cost-effectiveness, QALY

## Abstract

**Background:**

The REVOLENS study compared lung volume reduction coil treatment to usual care in patients with severe emphysema at 1 year, resulting in improved quality-adjusted life-year (QALY) and higher costs. Durability of the coil treatment benefit and its cost-effectiveness at 2 years are now assessed.

**Methods:**

After one year, the REVOLENS trial’s usual care group patients received coil treatment (second-line coil treatment group). Costs and QALYs were assessed in both arms at 2 years and an incremental cost-effectiveness ratio in cost per QALY gained was calculated. The uncertainty of the results was estimated by probabilistic bootstrapping.

**Results:**

The average cost of coil treatment in both groups was estimated at €24,356. The average total cost at 2 years was €9655 higher in the first-line coil treatment group (*p* = 0.07) and the difference in QALY between the two groups was 0.127 (*p* = 0.12) in favor of first-line coil treatment group. The 2-year incremental cost-effectiveness ratio (ICER) was €75,978 / QALY. The scatter plot of the probabilistic bootstrapping had 92% of the replications in the top right-hand quadrant.

**Conclusion:**

First-line coil treatment was more expensive but also more effective than second-line coil treatment at 2 years, with a 2-year ICER of €75,978 / QALY.

**Trial registration:**

ClinicalTrials.gov Identifier NCT01822795.

## Background

Usual medical treatments have limited effectiveness in patients with severe emphysema, justifying the development of alternative interventional treatments such as endobronchial lung volume reduction treatments including valves, coils, and thermal vapor ablation [1]. Endobronchial coil treatment (ECT) consists on placing non-blocking shape-memory nitinol coils into subsegmental airways to reduce dynamic lung hyperinflation [2]. Three randomized studies demonstrated that ECT is associated with improvements in exercise capacity, lung function and quality of life [3–5]. Only one economic evaluation was carried out by the REVOLENS study (réduction volumique endobronchique par spirales; NCT01822795) [4]. The economic evaluation’s goal is to evaluate the joint distribution of costs and benefits in order to assess the efficiency of a new intervention and therefore determine whether it would be a good use of healthcare resources. As such, they inform decision-makers who decide if a new technology should be reimbursed. The REVOLENS study found a mean cost difference between groups at 1 year of €36,123 per patient (*p* < 0.001), a mean quality-adjusted life year (QALY) difference of 0.061 (*p* = 0.02) and an incremental cost-effectiveness ratio (ICER) of €590,079 per QALY [4]. However, the one-year duration of the follow-up precluded any robust conclusions regarding the long-term cost-effectiveness of ECT. In addition, it did not address the question of the benefit (or lack thereof) of an early ECT management. The aim of the REVOLENS-2 study was therefore to estimate the 2-year cost-effectiveness of first-line lung volume reduction coil treatment compared to second-line coil treatment in patients with severe emphysema included in the REVOLENS trial.

## Methods

### Study design and patients

The design of the REVOLENS trial has been previously reported and will be briefly summarized [4]. In this prospective randomized open blinded end-point trial conducted in ten French sites, patients with bilateral emphysema, post bronchodilator forced expiratory volume in one second (FEV_1_) < 50% pred., residual volume > 220% pred., and formal pulmonary rehabilitation within the previous 12 months, were randomly assigned in a 1:1 ratio to coils treatment or to usual care. This study was approved by the Ethics Committee of Dijon Est I (N°2012-A01477–36), and by the French Agency for Medicines and Health Products (ANSM). The coils were purchased from the manufacturer (PneumRx/BTG, Mountain View, CA) which had no involvement in the study design. All participants signed a written informed consent to participate to the study. The primary endpoint of the REVOLENS trial was the improvement of at least 54 m in the 6-min walk test at 6 months.

In the REVOLENS protocol, patients of the usual care group were offered coil treatment after one year. In the REVOLENS-2 study, this group of patients is referred to as the second-line coil treatment group and is compared to the first-line coil treatment group. According to the protocol, patients in both groups were followed during 5 years post treatment. During this follow-up period, medical data (e.g. intervention data, biological analysis, imaging, respiratory function tests results) and economic data (e.g. quality of life, hospitalizations and consultations) were collected prospectively in a Case Report Form (CRF).

### Economic evaluation

Data for the economic evaluation were prospectively collected during the trial, in accordance with the Consolidated Health Economic Evaluation Reporting Standards (CHEERS) statement [6]. Costs and QALYs were assessed in both arms at 2 years and an incremental cost-effectiveness ratio in cost per QALY gained was calculated. The uncertainty of the results was analyzed using a non-parametric bootstrap which provided multiple estimates of the ICER by randomly re-sampling the patient population 1000 times and results were presented as a scatter plot of 1000 ICERs on the cost-effectiveness plane and transformed into a cost-effectiveness acceptability curve based on the decision-makers’ willingness to pay for an additional QALY. The analysis was conducted from the French healthcare perspective (mandatory health insurance, complementary health insurance and patient co-payments) using tariffs and hospital production costs for coil treatment. The time horizon was 2 years.

### Costs

Only direct costs were included as recommended by the French National Authority for Health (HAS) [7]. Both hospital and non-hospital resources were considered.

Initial hospitalization and monitoring data were obtained from the CRF and from the local hospital claims database. In addition, for interventional procedure data, a bottom-up micro-costing based on 5 French participating hospital visits was carried out.

The number and type of staff and medical devices required for the procedure (coils, delivery catheter, mucus vacuum cleaner and antibiotic therapy), procedures’ duration, the type of operating room and the type and length of hospital stay but also, the number and type of consultations (anesthesiologist, general practitioner, pulmonologist and physiotherapist), biology act (microbiological analysis of endobronchial aspirates), imaging (chest X-ray, chest computed tomography), medication (long term oxygen therapy), monitoring tests (walk test, blood gas test and respiratory function tests), transportation and readmission were all used as variables to calculate costs.

Staff costs per hour were calculated using the average salary costs which includes charges for each type of staff for full-time contracts of 1607 h per year [8]. Medical devices costs were valued by the manufacturer price. Consultations, laboratory tests, imaging, medication, monitoring tests and transportation costs were valued using the statutory health insurance tariffs [9–13]. Operating room costs were derived from the cost accounting of an operating room in one hospital visited. Hospital stays were valued using the French national hospital cost study (Etude Nationale de Coûts à méthodologie Commune, ENCC) adjusted to the length of stay observed in the study [14]. Readmissions costs were valued using the tariff of the corresponding diagnosis related groups [15] to which were added intensive care daily supplements [16].

Unit costs are presented in eTable 1 of the REVOLENS clinical trial article. Since 2017, the price of coils decreased from €1424 to €1080 per unit with a maximum of 10 coils charged per treated lobe. The current price for the economic evaluation was used. All other costs were in 2016 Euros (€) or inflated to 2016 using the health-specific inflation index [17]. A discount rate of 4% was applied to both outcomes and costs beyond the first year [7].

### Effectiveness

The effectiveness was expressed as the difference in QALYs during the 2-years follow-up period between the two arms. QALY represents a patient’s survival time weighted by the quality of life, represented by utility. Utility values were collected in the CRF from the EQ-5D-5 L health-related quality of life questionnaire. The EQ-5D-5 L comprises a descriptive system which is composed of five health dimensions (mobility, self-care, usual activities, pain/discomfort and anxiety/depression) with 5 levels of health state (no problems to extreme problems). The participant’s answers are combined to produce a five-digit number describing the participant’s health status which is converted to a utility value from the country specific value set. The French EQ-5D-5 L value set has utility between − 0.530 (worst possible health) and 1 (best possible health) [18–20].

### Statistical analysis

The statistical analyses were performed on the intention-to-treat (ITT) population. Qualitative data were presented using frequencies (percentages) and compared with the Chi-square test. Quantitative data were presented using means (standard deviations) and compared using Student t test or Mann-Whitney test depending on the variables’ distribution. Missing data were imputed either by the average in the case of quantitative variables or by the weighted frequency in the case of qualitative variables. A *p*-value less than 0.05 was considered significant. SAS (Version 9.3, SAS Institute, Cary, NC, USA) was used for analysis.

## Results

### Patients and procedures

**Fig. 1:**
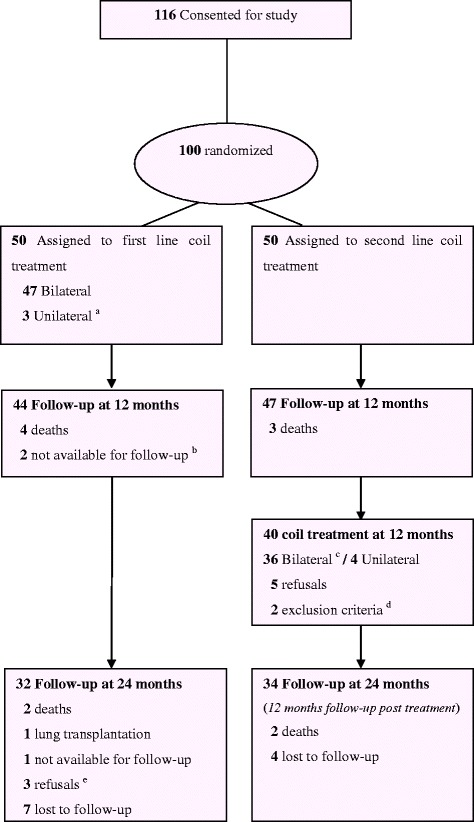
Flow chart of the study (CONSORT). ^a^ The reasons for not performing bilateral treatment were death before second treatment (*n*=1), anaphylactic shock at induction of anesthesia for the second coil treatment (*n*=1) (further analyses demonstrated allergy to penicillin), and pneumonia after the first coil treatment leading to unwillingness of the patient to undergo a second coil treatment (*n*=1). Two patients with unilateral coil treatment at 3-month follow-up were treated with a contralateral coil treatment at 12 and 18 months post-randomisation. ^b^ These two patients were alive at 12 months, but did not come for the planned visit at 12 months, and were considered for subsequent assessment at 24 months. ^c^ Bilateral treatment not performed because of pneumothorax (*n*=1), pneumonia (*n*=2) or death (*n*=1). One patient was treated with a contralateral coil treatment at 27 months post first treatment. ^d^ One systolic pulmonary artery pressure > 50 mmHg and one anticoagulant therapy which could not be stopped for coil treatment and also active smoking.^e^ 1 patient moved abroad and two patients refused to come back for the follow-up

Of 116 patients screened, 100 patients were randomized, 50 to the first line coil treatment group and 50 to the second line coil treatment group. In the first line coil treatment group, 47 patients received bilateral and 3 unilateral coil treatment, and in the second line coil treatment group, 36 patients received bilateral and 4 unilateral coil treatment. The flow chart of the study is presented in Fig. 1.

### Costs

The average length of stay for initial hospitalization was 3.1 days (± 1.6, min: 2, max: 10) for the first coil procedure and 3.6 days (± 2.5, min: 2, max: 13) for the second procedure in the first-line coil treatment group and 3.5 days (± 4.1, min: 2, max: 24) for the first coil treatment and 2.5 days (± 1.1, min: 2, max: 6) for the second procedure in the second-line coil treatment group. The mean coil treatment cost was estimated at €24,356 (9465). This cost was estimated at €27,800 (5629) in the first-line coil treatment group and at €20,912 (11,190) in the second-line coil treatment group (*p* = 0.04). The number of patients with at least one rehospitalization was 25 (50%) in the first-line coil treatment group and 26 (52%) in the second-line coil treatment group (*p* = 0.84). The average total cost per patient at 2 years was €9655 higher in the first-line coil treatment group (*p* = 0.07) (Table 1).

**Table 1:** Costs (inflated and discounted) in € by randomization group over a 2-year period

**Average cost per patient in € (SD)**	**First-line coil treatment group**	**Second-line coil treatment group**	***P*** **value**
**0–12 months after randomization**	***N*** **=** **50**	***N*** **=** **50**	
First coil procedure	14,412 (2358)	NA	*NA*
Second coil procedure (*N* = 47)	14,022(2471)	NA	*NA*
Rehospitalization	1486 (3352)	674 (1983)	*0.15*
Consultations	984 (1053)	987 (1209)	*0.24*
Transportation	265 (317)	121 (189)	*0.01*
Home oxygen	2222 (1925)	2040 (1933)	*0.49*
Monitoring tests	505 (72)	519 (36)	*0.29*
Imaging	125 (20)	105 (7)	*<.0001*
**Average total cost during the first year** ^**a**^	**33,388 (6949)**	**4446 (2644)**	***<.0001***
**12–24 months after randomization**	***N*** **= 46**^**b**^	***N*** **= 47**^**b**^	
First coil procedure (*N* = 40)	NA	14,022 (996)	*NA*
Second coil procedure (*N* = 36)	NA	13,465 (501)	*NA*
Rehospitalization	4912 (19,662)	2897 (4,4862)	*0.19*
Consultations	460 (630)	647 (1046)	*0.005*
Transportation	31 (48)	132 (159)	*0.002*
Home oxygen	2076 (1949)	1790 (1887)	*0.51*
Monitoring tests	97 (21)	97 (21)	*0.44*
Imaging	20 (4)	57 (18)	*<.0001*
**Average total cost during the second year for patients alive** ^**a**^	**7596 (20,035)**	**27,867 (12,487)**	***<.0001***
**0–24 months after randomization**	***N*** **= 50**	***N*** **= 50**	
Total cost for 50 patients per group^c^	2,018,781	1,536,027	*NA*
**Average total cost per patient**	**40,376 (21,173)**	**30,721 (14,364)**	***0.07***

^a^Patients who did not have the coil treatment have estimated costs of €0

^b^Patients alive at 12 months

^c^The ITT population. Patients who died or did not have the coil treatment have estimated costs of €0

The bold datas are number of patients, subtotal and total

### Effectiveness

**Fig. 2:**
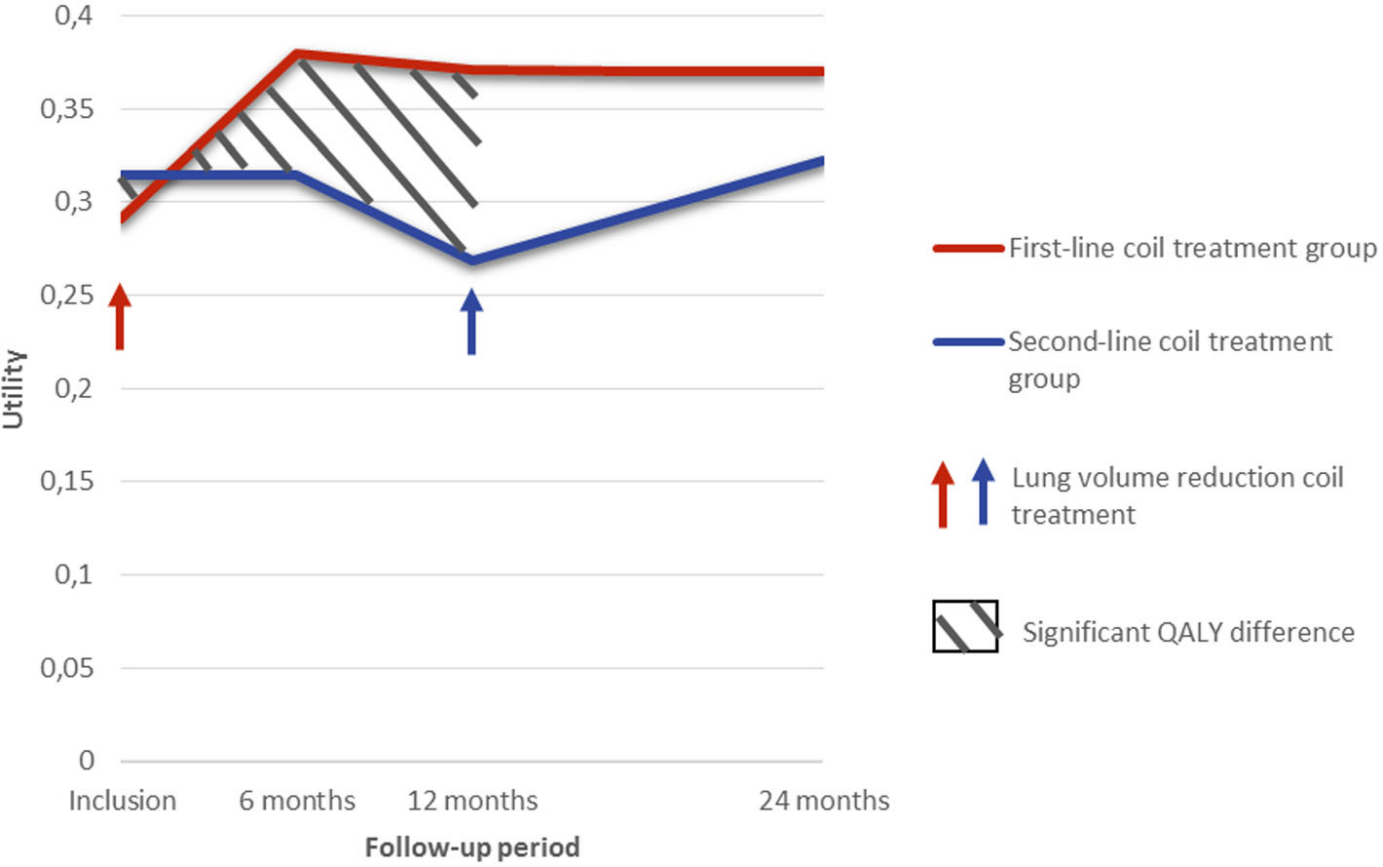
Utilities during the 2-years follow-up period for both groups. The total QALYs in each group are represented by the area under the curves and the QALY difference between the 2 groups by the area between the curves

The average QALY at 2 years was 0.726 (0.400) in the first-line coil treatment group versus 0.599 (0.406) in the second-line coil treatment group. The difference in QALY between the two groups was 0.010 at 6 months (*p* = 0.62), 0.061 at 1 year (*p* = 0.02) and 0.127 at 2 years (*p* = 0.12). Utilities during the 2-years follow-up period for both groups are presented in Fig. 2. The total QALYs in each group are represented by the area under the curves and the QALY difference between the 2 groups by the area between the curves. There was an improvement in the post-treatment quality of life in both groups. However in the second-line coil treatment group, quality of life significantly decreased during the first year (pre-treatment) and did not increase to the level of the first-line coil treatment group’s quality of life after treatment.

### ICER

**Fig. 3:**
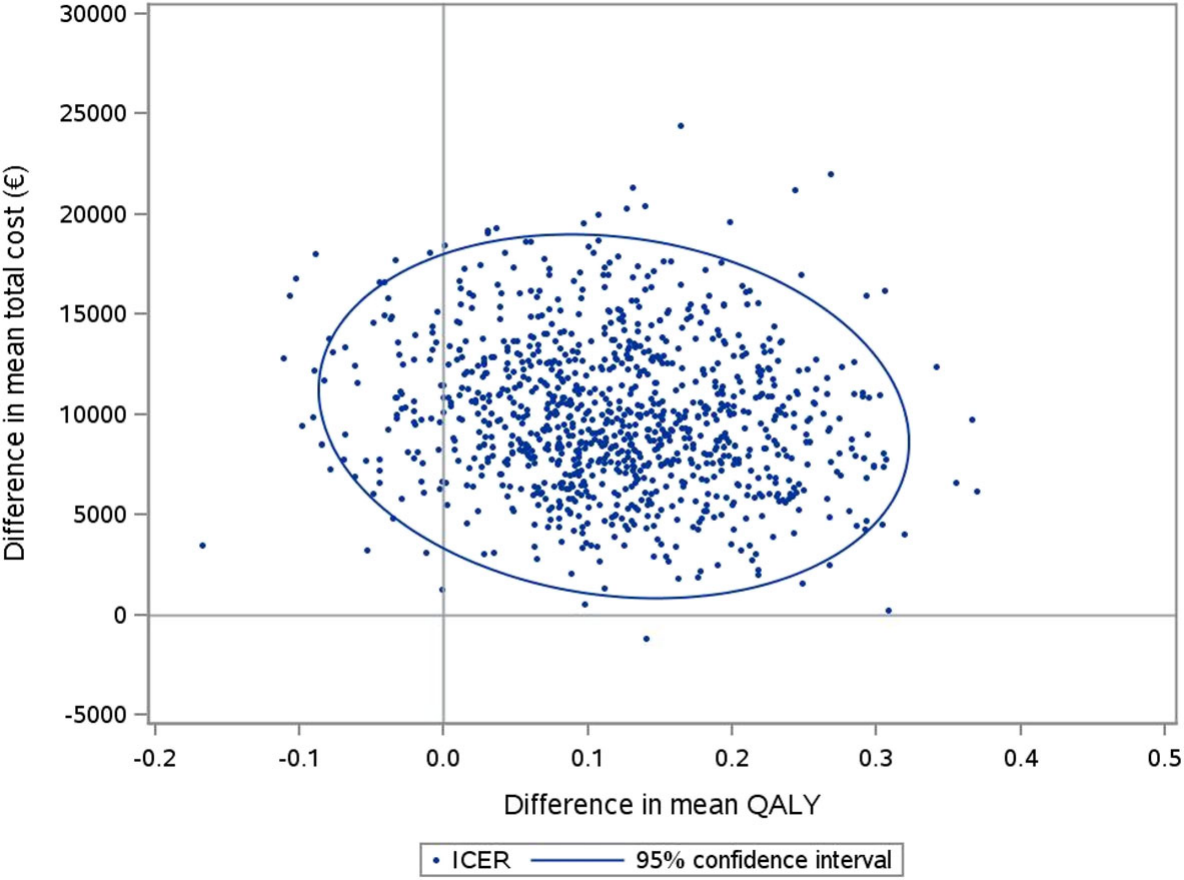
Scatter plot of incremental cost and effectiveness of first-line coil treatment compared to second-line coil treatment The set of ICERs estimated by the non-parametric bootstrap are presented by the cloud of points on the cost-effectiveness plane.

**Fig. 4:**
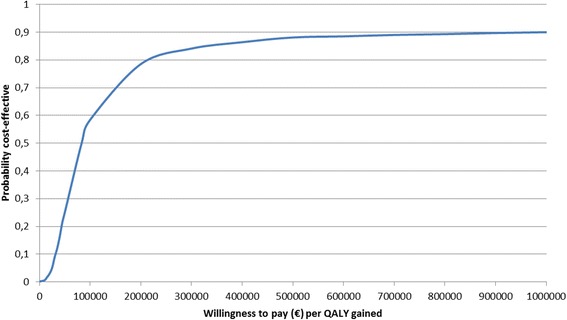
Cost-effectiveness acceptability curve showing the probability that first-line coil treatment is cost-effective compared to second-line coil treatment

The 2-year ICER was €75,978 / QALY. The set of ICERs estimated by the non-parametric bootstrap are presented by the cloud of points on the cost-effectiveness plane. 92% of these ICERs were located in the top right-hand quadrant, indicating a higher cost for greater effectiveness of first-line coil treatment (Fig. 3). In addition, the acceptability curve showed that at a threshold of €83,200 / QALY there was 50% chance that the first-line coil treatment was cost-effective (Fig. 4).

## Discussion

This health economic analysis of the REVOLENS study results was prospectively designed to inform healthcare payers in France.

The results showed that the mean 2-year treatment cost was estimated at €27,800 per patient in the first-line coil treatment group and at €20,912 in the second-line coil treatment group. Patients in the second-line treatment group received coil treatment at 1 year and the cost difference between the two groups decreased from €28,941 at 1 year to €9655 at 2 years. The non-significant €9655 cost difference between the 2 groups was due to rehospitalization and coil treatment costs which tended to be higher in the first-line coil treatment group. This can be explained by the fact that only 40 out of 50 patients had the procedure. The cost of the intervention was thus zero euro for the remaining 10 patients.

Furthermore, there was an improvement in the post-treatment quality of life in both groups. However in the second-line coil treatment group, quality of life significantly decreased during the first year (pre-treatment) and did not increase to the level of the first-line coil treatment group’s quality of life at one year after coil treatment, suggesting that early lung volume reduction coil treatment might be associated with better quality of life outcome.

The decrease in incremental cost and the increase in incremental QALY had led to an ICER of €75,978 / QALY at 2 years compared to an ICER of €472,759 / QALY at 1 year. The difference between the 1-year ICER of the REVOLENS-2 study and the 1-year ICER of the primary economic evaluation (€590,079 / QALY) was explained by the lower price of coils and 2015/2016 inflation.

The probabilistic bootstrapping, by graphically demonstrating an ICER variation when resampling the patients 1000 times, showed a large dispersion, resulting from the uncertainty surrounding the clinical results. 92% of the replications were in the top right-hand quadrant, indicating a higher cost for greater effectiveness of fist-line coil treatment.

These results provide important data for healthcare payers appraising the cost-effectiveness of ECT in severe emphysema. Indeed, literature regarding cost-effectiveness in endobronchial lung volume reduction is scarce, limited to a model-based assessment of valves [21], showing an estimated 5-year ICER of €46,322 / QALY and a 10-year of €25,142 / QALY. The REVOLENS study is focused on ECT and was carefully designed to overcome the methodological shortcomings of model-based assessment. Estimation of the 2-year ICER of endobronchial coils is based on rigorous methodology from prospective data and following the international standards of health-economic assessments which allows this study to have a high internal validity. However the external validity is more moderate because of the high cost of the medical device and the variability of the operators’ skills.

The time horizon chosen for the economic evaluation is 2 years. Because patients of the usual care group were offered coil treatment after one year of follow-up, it can be assumed that the difference in cost between the two groups remained unchanged and the difference in utility either remained unchanged or vanished after the second year of follow-up. In the first case, the stabilization of the utility 1 year after the coil treatment in the first-line coil treatment group suggests there could be a similar stabilization in the second-line coil treatment group after year 2. At 5 years, the utility difference between the 2 groups present at 2 years could therefore be maintained. Likewise, the cost difference after the second year of follow-up has no reason to change based on the non-significant difference between the 2 groups in rehospitalizations and home oxygen costs during the second year of follow-up, which are the 2 major costs. As such, we would expect the incremental cost-effectiveness ratio at 5 years to be lower than the ICER at 2 years, making early treatment more cost-effective in the long-term. In the latter case, the ICER would also decrease, but not as much. An 18 month utility measurement would have refined our assumption. In order to answer this question, data at 5 years will be available soon but it might be challenging to obtain hospital data in the long term. The PneumRx endobronchial coil system in treatment of subjects with severe emphysema study (ELEVATE study) will start in 2018. This international study aims to improve patient selection in order to reduce the number needed to treat and improve the cost effectiveness [22].

Finally, there is limited availability of this technology across the world but due to the lack of effective therapies for patients with severe emphysema, studies on endobronchial lung volume reduction are of interest for pulmunologists internationally.

## Conclusion

In conclusion, REVOLENS-2 study results showed that first-line coil treatment was more expensive but also more effective than second-line coil treatment at 2 years, with a 2-year ICER of €75,978 / QALY.

## References

[1] Shah PL, Herth FJ, van Geffen WH, Deslee G, Slebos D-J (2017). Lung volume reduction for emphysema. Lancet Respir Med.

[2] Makris D, Leroy S, Pradelli J, Benzaquen J, Guenard H, Perotin J-M, Zakynthinos S, Zakynthinos E, Deslee G, Marquette CH. Changes in dynamic lung mechanics after lung volume reduction coil treatment of severe emphysema. Thorax 2017. 10.1136/thoraxjnl-2017-210118. 10.1136/thoraxjnl-2017-210118 28893857

[3] Shah PL, Zoumot Z, Singh S, Bicknell SR, Ross ET, Quiring J, Hopkinson NS, Kemp SV, RESET trial Study group. Endobronchial coils for the treatment of severe emphysema with hyperinflation (RESET): a randomised controlled trial. Lancet Respir Med. 2013; 1: 233–240. 10.1016/S2213-2600(13)70047-X 24429129

[4] Deslée G, Mal H, Dutau H, Bourdin A, Vergnon JM, Pison C, Kessler R, Jounieaux V, Thiberville L, Leroy S, Marceau A, Laroumagne S, Mallet JP, Dukic S, Barbe C, Bulsei J, Jolly D, Durand-Zaleski I, Marquette CH, REVOLENS Study Group (2016). Lung volume reduction coil treatment vs usual Care in Patients with Severe Emphysema: the REVOLENS randomized clinical trial. JAMA.

[5] Sciurba FC, Criner GJ, Strange C, Shah PL, Michaud G, Connolly TA, Deslée G, Tillis WP, Delage A, Marquette C-H, Krishna G, Kalhan R, Ferguson JS, Jantz M, Maldonado F, McKenna R, Majid A, Rai N, Gay S, Dransfield MT, Angel L, Maxfield R, Herth FJF, Wahidi MM, Mehta A, Slebos D-J, RENEW Study Research Group (2016). Effect of endobronchial coils vs usual care on exercise tolerance in patients with severe emphysema: the RENEW randomized clinical trial. JAMA.

[6] Husereau D, Drummond M, Petrou S, Carswell C, Moher D, Greenberg D, Augustovski F, Briggs AH, Mauskopf J, Loder E (2013). ISPOR health economic evaluation publication guidelines-CHEERS good reporting practices task force. Consolidated health economic evaluation reporting standards (CHEERS)--explanation and elaboration: a report of the ISPOR health economic evaluation publication guidelines good reporting practices task force. Value Health J Int Soc Pharmacoeconomics Outcomes Res.

[7] Haute Autorité de Santé. Choix méthodologiques pour l’évaluation économique. [Internet]. [cited 2014 Oct 1].Available from: http://www.has-sante.fr/portail/upload/docs/application/pdf/2011-11/guide_methodo_vf.pdf.

[8] Guide pour le suivi de la masse salariale [Internet]. Direction générale de l’offre de soins; 2014.Available from: http://solidarites-sante.gouv.fr/IMG/pdf/dgos_guide_suivi_masse_salariale_2014.pdf.

[9] Assurance Maladie. Les consultations en métropole. [Internet]. Ameli.fr [cited 2017 Feb 1].Available from: http://www.ameli.fr/assures/soins-et-remboursements/combien-serez-vous-rembourse/consultations/les-consultations-en-metropole/dans-le-cadre-du-parcours-de-soins-coordonnes_rhone.php.

[10] Assurance Maladie. Classification commune des actes médicaux. [Internet]. [cited 2017 Feb 1].Available from: http://www.ameli.fr/accueil-de-la-ccam/index.php.

[11] Assurance Maladie. Table nationale de codage de biologie. [Internet]. [cited 2017 Feb 1].Available from: http://www.codage.ext.cnamts.fr/codif/nabm/index_presentation.php?p_site=AMELI.

[12] Eco-santé 2013 [Internet]. IRDES [cited 2015 May 5]. Available from: http://www.ecosante.fr/index2.php?base=DEPA&langh=FRA&langs=FRA.

[13] Base des médicaments et informations tarifaires [Internet]. [cited 2013 Oct 23].Available from: http://www.codage.ext.cnamts.fr/codif/bdm_it/index_presentation.php?p_site=AMELI.

[14] Référentiel de coûts MCO 2014 [Internet]. Scan Santé [cited 2016 Dec 9].Available from: http://www.scansante.fr/r%C3%A9f%C3%A9rentiel-de-co%C3%BBts-mco-2014.

[15] ATIH. Tarifs MCO et HAD. [Internet]. [cited 2017 Feb 1].Available from: http://www.atih.sante.fr/tarifs-mco-et-had.

[16] Tarification de référence [Internet]. ATIH [cited 2016 Sep 10].Available from: http://www.atih.sante.fr/tarification-de-reference.

[17] Ministère des Solidarités et de la Santé. Les dépenses de santé en 2016 - Résultats des comptes de la santé [Internet]. Ministère Solidar. Santé 2017 [cited 2018 Jan 15].Available from: http://drees.solidarites-sante.gouv.fr/etudes-et-statistiques/publications/panoramas-de-la-drees/article/les-depenses-de-sante-en-2016-resultats-des-comptes-de-la-sante-edition-2017.

[18] Nolan CM, Longworth L, Lord J, Canavan JL, Jones SE, Kon SSC, Man WD-C (2016). The EQ-5D-5L health status questionnaire in COPD: validity, responsiveness and minimum important difference. Thorax.

[19] EQ-5D-5L – EQ-5D [Internet]. Euroqol [cited 2017 Jun 23].Available from: https://euroqol.org/eq-5d-instruments/eq-5d-5l-about/.

[20] Gusi N, Olivares PR, Rajendram R. The EQ-5D Health-Related Quality of Life Questionnaire. In: Preedy VR, Watson RR, editors. Handb. Dis. Burd. Qual. Life Meas. [Internet] Springer New York; 2010 [cited 2017 Jun 23]. p. 87–99Available from: http://link.springer.com/referenceworkentry/10.1007/978-0-387-78665-0_5.

[21] Pietzsch JB, Garner A, Herth FJF (2014). Cost-effectiveness of endobronchial valve therapy for severe emphysema: a model-based projection based on the VENT study. Respir Int Rev Thorac Dis.

[22] Study of PneumRx Endobronchial Coil System in Treatment of Subjects With Severe Emphysema (ELEVATE) [Internet]. ClinicalTrial.gouv [cited 2018 Feb 15].Available from: https://clinicaltrials.gov/ct2/show/NCT03360396.

